# Influência da Genética no Desenvolvimento da Hipertensão

**DOI:** 10.36660/abc.20230755

**Published:** 2024-02-16

**Affiliations:** 1 Universidade Federal do Rio Grande do Sul Faculdade de Medicina Porto Alegre RS Brasil Programa de Pós-Graduação em Cardiologia – Faculdade de Medicina – Universidade Federal do Rio Grande do Sul, Porto Alegre, RS – Brasil

**Keywords:** Hipertensão, Genética, Brasil

A hipertensão arterial sistêmica primária é uma das doenças mais comuns em todo o mundo, estimando-se que afete 20% das mulheres e 25% dos homens.^1^ É um fator de risco para metade das dez principais causas de morte, incluindo a líder do ranking, doença cardiovascular, seguida de doença cerebrovascular e renal.^2^ Há muitos anos, grandes esforços vêm sendo feitos para desvendar os mecanismos que contribuem para esta condição que - apesar de silenciosa - pode ser devastadora. Alguns advogam que a hipertensão não é uma doença, e sim uma síndrome, que apresenta etiologia individual variada e tem como sinal comum a elevação da pressão arterial.^3^ Alguns fatores previamente associados à hipertensão primária já são bem estabelecidos, por exemplo obesidade, tabagismo, desequilíbrio do sistema renina-angiotensina-aldosterona (SRAA), estresse mental, entre outros.^4^ Os fatores genéticos, no entanto, têm influência vaga e complexa no desenvolvimento da hipertensão. Apesar de vários estudos dedicados ao esclarecimento do o assunto, seu caráter poligênico torna cada caso único no que diz respeito ao impacto de cada fator predisponente. Vários polimorfismos em diferentes genes foram apontados como correlacionados com hipertensão,^5^ incluindo o polimorfismo C825T da subunidade β3 da proteína G (GNB3) (rs5443:C>T). As proteínas de ligação a nucleotídeos guanina (proteínas G) são expressas na maioria dos tecidos humanos e funcionam como transdutores em vias de sinalização intracelular, regulando uma ampla variedade de processos fisiológicos. O alelo 825T foi previamente associado à hipertensão e à obesidade; no entanto, alguns estudos foram controversos e as frequências do alelo T diferiram entre diferentes etnias (fT≈0,8 em africanos negros, fT≈0,45 em asiáticos e fT≈0,3 em brancos).^6^

O estudo de Agostini et al.^7^ teve como objetivo caracterizar uma amostra de 155 hipertensos brasileiros para avaliar a prevalência do polimorfismo C825T e correlacioná-lo com características clínicas.^7^ Diferente da maioria dos estudos,^8^ o grupo não encontrou associação entre a genética variante e hipertensão. A identificação e seleção dos hipertensos basearam-se exclusivamente em dados de entrevistas em prontuários (uso ou não de medicamento anti-hipertensivo e registro ou não registro de diagnóstico prévio), o que pode ter interferido em seus achados. Além disso, foi demonstrado anteriormente que a frequência do alelo T pode variar entre as etnias, assim como a prevalência de hipertensão. Dessa forma, embora a ideia de caracterizar geneticamente a população brasileira como uma unidade seja interessante, a população brasileira é uma das mais heterogêneas do globo. Portanto, olhar paralelamente para os grupos étnicos de forma separada, poderia enriquecer a análise. O perfil metabólico também foi comparado entre os grupos e encontrou associações positivas de hipertensos apresentando o polimorfismo com níveis mais elevados de triglicerídeos, glicose, ácido úrico e obesidade, semelhantes a relatos anteriores. A elucidação das vias da hipertensão é um assunto muito complexo, especialmente no que diz respeito à genética, pelo que cada esforço para identificar fatores predisponentes acrescenta uma nova peça valiosa a este quebra-cabeças.

A conscientização sobre o valor da avaliação genética na assistência médica no Brasil ainda está longe do ideal em diversas áreas, inclusive na cardiologia. Além da barreira do seu alto custo, existe a ideia comum de que o perfil genético não gera impacto significativo no manejo clínico. Ainda que a pesquisa caminhe frequentemente com pequenos passos, toda contribuição é de extrema importância e deve ser incentivada. O grupo de Agostini abordou uma das condições mais impactantes do mundo e trouxe grande conhecimento do ponto de vista genético – um campo no qual pesquisadores e profissionais de saúde brasileiros estão em fase de desenvolvimento e necessitam incentivo para explorar. Nesse contexto, independente dos possíveis achados finais, seu estudo significa contribuição valiosa e inovadora para a comunidade científica brasileira.
