# Supplementary material for: Influência da Genética no Desenvolvimento da Hipertensão
Source: Arq Bras Cardiol. 2024 Feb 16;120(12):e20230755. [Article in Portuguese] doi: 10.36660/abc.20230755 (PMC11021032; doi:10.36660/abc.20230755)
Supplement: Translation

# Translation: Influence of Genetics on Hypertension Development

**Affiliations:** 1 Porto Alegre RS Brazil Programa de Pós-Graduação em Cardiologia – Faculdade de Medicina – Universidade Federal do Rio Grande do Sul, Porto Alegre, RS – Brazil

**Keywords:** Hypertension, Genetics, Brazil

Primary hypertension is one of the most common diseases worldwide, estimated to affect 20% of females and 25% of males.^1^ It is a risk factor for half of the ten leading causes of death globally, including the ranking leader, cardiovascular disease, and right next to it, brain, and kidney disease.^2^ For a long time, major efforts have been made to unravel the mechanisms that contribute to this - mostly – silent but devastating condition. Some advocate that hypertension is not a disease but a syndrome; a condition with multiple etiological factors which have as common sign an elevated blood pressure.^3^ Amongst intrinsic and environmental factors related to primary hypertension, some are already well established, e.g., obesity, smoking, disbalance of the renin-angiotensin-aldosterone-system (RAAS), mental stress, etc.^4^ The role of genetics in this scenario, nevertheless, has a vague and complex-to-characterize influence on hypertension development. Despite several studies dedicated to enlighten the matter, its polygenic nature makes every case unique regarding the impact of each predisposing factor. Several polymorphisms in different genes were pointed to correlate with hypertension,^5^ including the G-protein β3 subunit (GNB3) C825T polymorphism (rs5443:C>T). Guanine nucleotide-binding proteins (G proteins) are expressed in most human tissues and function as transductors in intracellular signaling pathways, regulating a wide variety of physiological processes. The 825T allele was previously associated with hypertension and obesity; however, a few studies were controversial, and the frequencies of the T allele differed amongst different ethnicities (fT≈0,8 in black Africans, fT≈0,45 in Asians, and fT≈0,3 in whites).^6^

The study by Agostini et al.^7^ aimed to characterize a sample of 155 hypertensive Brazilian individuals in order to assess the prevalence of the C825T polymorphism and correlate it with clinical features.^7^ Unlike most of the other studies,^8^ the group found no association between the genetic variant and hypertension. The identification and selection of hypertensive subjects were based solely on interview data from medical records (use or not of anti-hypertensive medication and register or lack of register of a previous diagnosis), which might have interfered with their findings. Also, it was shown previously that the frequency of the T allele between ethnicities is often not equal, as well as hypertension prevalence. Thus, even though the idea of genetically characterizing the Brazilian population as a unity can be interesting, the Brazilian population is one of the most heterogeneous in the globe. Therefore, looking at ethnical groups separately could have enriched the analysis. The metabolic profile was also compared between groups and found positive associations of hypertensive individuals presenting the polymorphism with higher levels of triglycerides, glucose, uric acid, and obesity, similar to previous reports. The elucidation of hypertension pathways is a very complex subject, especially regarding genetics, so every effort to identify predisposing factors adds a new valuable piece to this puzzle.

The awareness about the value of genetic assessment in medical care in Brazil is still far from ideal in several areas, including cardiology. Besides the barrier of its high cost, there is the common idea that genetic profiling will not have a significant impact on clinical management. Even though research frequently walks with small steps, every contribution is of extreme importance and must be encouraged. The group of Ms. Agostini approached one of the most impacting conditions worldwide and brought great insight into the genetics point of view - a field in which Brazilian researchers and health care professionals are still developing and need incentive to explore. In this context, regardless of any possible outcome, their study mean a valuable and innovative contribution to Brazilian scientific development.
